# Assessment of Serum Vascular Endothelial Growth Factor Levels in Pregnancy-Induced Hypertension Patients

**DOI:** 10.1155/2017/3179670

**Published:** 2017-01-04

**Authors:** Vibha Tandon, Swati Hiwale, Dnyanesh Amle, Tripti Nagaria, Pradeep Kumar Patra

**Affiliations:** ^1^Department of Biochemistry, Pt. J.N.M. Medical College and Dr. B.R.A.M. Hospital, Raipur, Chhattisgarh, India; ^2^Department of Obstetrics and Gynaecology, Pt. J.N.M. Medical College and Dr. B.R.A.M. Hospital, Raipur, Chhattisgarh, India

## Abstract

*Objective*. The objective of the study was to assess the serum vascular endothelial growth factor (VEGF) levels in peripheral blood of patients with pregnancy-induced hypertension (PIH) and find association between serum VEGF levels and PIH.* Methods*. Thirty-five PIH subjects, 35 normal pregnant females, and 20 normal healthy females were included in the study. Detailed history, clinical examination, and relevant biochemical parameters were assessed; serum VEGF levels were estimated using Double-antibody enzyme-linked immunosorbent assay.* Results*. The study groups were found to be age matched (*p* = 0.38). VEGF level in the pregnancy-induced hypertensive group (median = 109.19 (3.38 ± 619)) was significantly higher than the normal pregnant (median = 20.82 (1.7–619)) and control (median = 4.92 (1.13–13.07)) group and the difference between these three groups was significant (*p* < 0.0001). The 3 groups are found to be significantly different in terms of RBS (*p* = 0.01), urea (*p* < 0.0001), creatinine (*p* = 0.0005), AST (*p* = 0.0032), ALT (*p* = 0.0007), total protein (*p* = 0.0004), albumin (*p* < 0.0001), calcium (*p* = 0.001), and sodium (*p* = 0.02), while no statistically significant difference was found between total bilirubin (*p* = 0.167), direct bilirubin (*p* = 0.07), uric acid (*p* = 0.16), and potassium (*p* = 0.14).* Conclusion*. Significantly higher levels of serum VEGF were noted in PIH subjects compared to normal pregnant and control subjects.

## 1. Introduction

Pregnancy-induced hypertension (PIH), especially preeclampsia, is the most common cause of the maternal and fetal morbidity and mortality in the developed and developing countries [1, 2]. Despite thorough studies by many authors, the exact pathophysiological mechanism behind this condition remains an enigma [3]. It complicates 5% to 7% of low risk pregnancies but the incidence of disease may rise up to 20% in high-risk pregnancies [4, 5]. PIH is a multiorgan disorder of complex vascular pathophysiology but yet incompletely understood etiology, which damage the brain, heart, kidney, and other organs. The pathophysiology of the disease progresses through combination of genetic, immunological, and environmental factors. Preeclampsia, though asymptomatic in early stage, is characterized by placental abnormality during the first trimester with consequent placental insufficiency and release of placental materials or cytokines into the maternal circulation. In the later stage of pregnancy, hypertension, proteinuria, hemolysis, raised liver enzymes, and low platelets are encountered and eventually eclampsia ensues [3–5].

Vascular endothelial growth factor (VEGF) [6], also termed as vascular permeability factor (VPF) [7], is a homodimeric (34–42 KDa), heparin-binding glycoprotein with proved role as potent angiogenic, mitogenic, and vascular permeability-enhancing activities. The expression of VEGF has been found in various tissues including activated macrophages [8], keratinocytes [9], glomerular visceral epithelium [10], hepatocytes [11], aortic smooth muscle cells [12], and embryonic fibroblasts [13]. The principle tissues expressing VEGF are surface of placental syncytiotrophoblast cells and invasive chorionic trophoblast cells during pregnancy with, in particular, expression at the vascular bud site during early pregnancy, when syncytiotrophoblast cells are abundant [14–17]. Overexpression of VEGF is responsible for vascular endothelial proliferation which may culminate in endothelial damage in long term.

The present study was aimed at investigating serum VEGF levels in PIH with special emphasis on finding a correlation between the two of them if any exists.

## 2. Materials and Methods

### 2.1. Design and Setting

This hospital based observational analytical study with cross-sectional data collection was carried out in Department of Biochemistry, Pt. J.N.M Medical College, Raipur, Chhattisgarh, in collaboration with Department of Obstetrics and Gynaecology, Pt. J.N.M Medical College, Raipur, Chhattisgarh. Institutional ethics committees of Pt. J.N.M Medical College, Raipur, Chhattisgarh, approved the study protocol. Institutional ethics committees of Pt. J.N.M Medical College, Raipur, Chhattisgarh, approved the study protocol. Informed written consent was obtained from all study participants.

### 2.2. Participants

The study population comprised of three groups,* Group 1*: 20 randomly selected cases of normal nonpregnant women with average age between 20 and 30 years,* Group 2*: 35 randomly selected cases of normal healthy pregnant women of gestational age between 24 and 36 weeks with average age between 20 and 30 years admitted in the hospital during the same period, and* Group 3*: 35 PIH women of gestational age between 24 and 36 weeks with average age between 20 and 30 years. Patients with hypertension, heart disease, diabetes mellitus, and liver and kidney diseases were excluded.

### 2.3. Data Collection

After obtaining informed written consent from the study participants, detailed history including that of chief complaint with onset, duration, and progress of current condition and obstetric history were elicited. Five mL of venous blood was drawn from anterior cubital vein by venepuncture after overnight fasting in a red vacutainer® (BD™ Biosciences) and was allowed to clot and then centrifuged for ten minutes at 4,000 rpm to obtain the serum. Biochemical analysis including renal function tests, liver function tests, and other routine biochemical parameters were analysed immediately on I Lab 650® clinical chemistry analyser (Werfen®, Germany) using manufacturer guidelines and around 1–1.5 mL of serum was stored in aliquots at –70°C for further estimation of serum VEGF by ELISA. Urinary protein was assayed by dipstick method (Mission® Urinalysis reagent strips, ACCH Biotech Hangzhou China Cat. number U031-021). Serum VEGF levels were assessed by double sandwich ELISA method using Novex® Human VEGF ELISA kit (Life Technology® Cat. number KHG0111).

### 2.4. Statistical Analysis

All statistical analysis was performed using SPSS version 16 (IBM Corp Ltd.). Normal distribution of the data for each parameter was checked by Kolmogorov-Smirnov analysis. Percentage, mean, and standard deviation were used to express normally distributed variables and median and range was used to express nonparametric variables. Student's *t*-test Wilcoxon Signed Rank test were performed to calculate the *p* value. *p* value < 0.05 was considered as statistically significant. ANOVA followed by post hoc Tukey's HSD test for parametric variables and Kruskal-Wallis test followed by post hoc Dunn test for nonparametric data were used to assess significance between more than two parameters. ROC curve was plotted to check the diagnostic significance of VEGF for PIH.

## 3. Result

We assessed 35 subjects each of PIH and normal pregnancy and a healthy control group including 20 females.  Table 1 indicates the general characteristics of the subjects in the study. All the three groups (PIH, normal pregnant, and healthy control group) were matched for age (*p* = 0.38). While no significant difference was noted in the terms of T. Bil (*p* = 0.16), D. Bil (*p* = 0.07), uric acid (*p* = 0.16), and serum potassium (*p* = 0.14) among three groups, significantly elevated levels of T. Bil (*p* < 0.05), D. Bil (*p* < 0.05), and potassium (*p* < 0.05) were noted in PIH group compared to control group. Also levels of uric acid were significantly higher in PIH group compared to normal pregnant but not control groups. Significant difference was noted in the terms of RBS (*p* = 0.01), urea (*p* < 0.0001), creatinine (*p* = 0.0005), AST (*p* = 0.0032), ALT (*p* = 0.0007), total protein (*p* = 0.0004), albumin (*p* < 0.0001), calcium (*p* = 0.001), and sodium (*p* = 0.02) among three groups.

**Figure 1:**
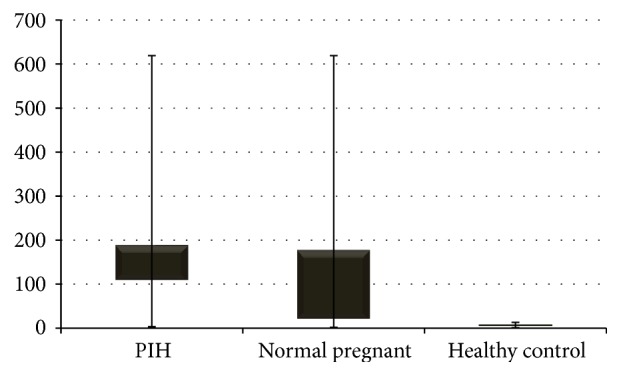
Comparison of serum VEGF level between PIH, normal pregnant, and control subjects.

**Figure 2:**
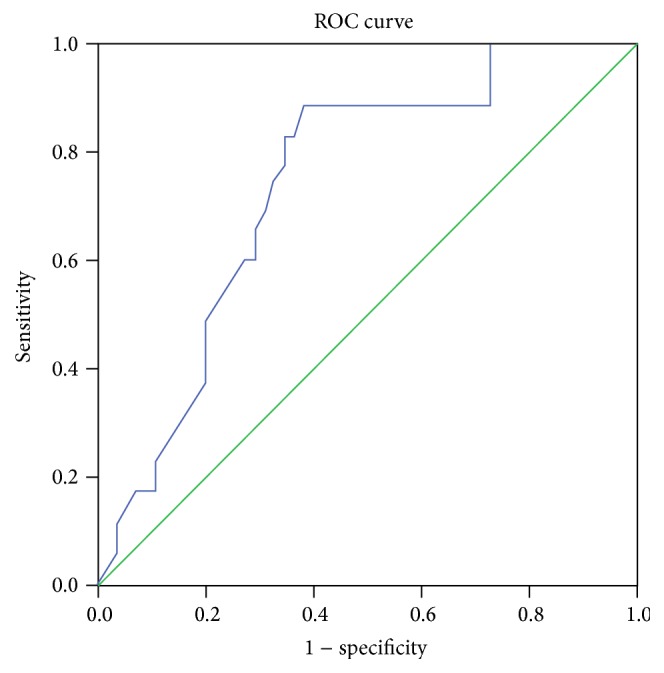
ROC curve was plotted to check the diagnostic significance of VEGF in PIH. Area under the curve was found to be 73.8% (*p* < 0.0001), 95% confidence interval range was 63.5–89%. At VEGF level 14.04 pg/mL, sensitivity was found to be 88.6% and specificity to be 61.8% (Youden's index *J* = 0.05). ROC plot demonstrates performance of serum VEGF to detect PIH. Diagonal segments are produced by ties.

In our study, we found a significant difference (*p* < 0.0001) in VEGF in comparison of PIH females, normal pregnant females, and normal nonpregnant females. The serum VEGF concentration median range in PIH (109.19 (3.38–619)) is higher than normal pregnant (20.82 (1.7–619)) and control (4.92 (1.13–13.07)) group; however, no significant difference existed between normal pregnant and control group (*p* > 0.05) (Figure 1, Table 2). ROC curve was plotted to check the diagnostic significance of VEGF in PIH. Area under the curve was found to be 73.8% (*p* < 0.0001), 95% confidence interval range was 63.5–89%, and at VEGF level was 14.04 pg/mL. Sensitivity was found to be 88.6% and specificity to be 61.8% (Youden's index *J* = 0.50) (Figure 2).

## 4. Discussion

In our study, we found an increased concentration of VEGF in PIH subjects compared to normal pregnant females and control group. In some recent studies, results have also shown that this elevated concentration of serum VEGF has increased to the level similar to that of the normal pregnant females without preeclampsia, leading authors to assume that the primary source of the elevated concentration of serum VEGF lies basically in the foetus and the placenta and with removal of the placenta and the foetus the level of serum VEGF will also revert to normal. According to previous studies in which different investigators have measured the VEGF concentration in the maternal circulation during normal and preeclamptic patients, results were conflicting with variable outcomes. Baker et al. [18] measured the concentration of VEGF in the preeclamptic, normal pregnant, and nonpregnant females by using an immunofluorescent ELISA assay; they have shown the elevated level of VEGF in the preeclamptic subjects while the undetectable levels were found in the normal pregnant and nonpregnant females. Similarly, Sharkey et al. [19] and Kupferminc et al. [20] found the elevated levels of VEGF in preeclamptic women with the use of a VEGF competitive enzyme immunoassay, but Lyall et al. [21] found decreased level of serum VEGF in preeclamptic and normal pregnant compared to control group. Authors have assessed the VEGF levels using commercial ELISA kit. These findings were interestingly quite different from the other previous findings because VEGF has been supposed to play an important role in both the embryogenesis and placental formation; thus, the elevated levels are expected in the pregnant females.

Some recent studies have revealed the presence of VEGF receptor known as soluble Fms-like tyrosine kinase (sFLT-1) in the blood of pregnant females but not in nonpregnant females or males. Normally, this sFLT-1 is present in the cell membrane on endothelial and extravillous trophoblast. This receptor binds the VEGF with high affinity resulting in preventing the action of VEGF on vascular endothelial cells [22]. However, it is not known that why this sFLT-1 is present in the maternal circulation during pregnancy. Considering the facts that sFLT-1 has high binding affinity with VEGF, the resultant concentration of biologically active VEGF is difficult to be interpreted. This may serve as explanation to the discrepancy in results of various studies including ours. Yet, the significantly increased levels of VEGF in PIH compared to those in normal pregnant subjects suggest relative excess of VEGF in PIH. Further from prior knowledge of raised sFLT-1 in both the subjects [22], it can be deducted that relative concentration of biologically active VEGF may be higher in PIH subjects but it is difficult to give conclusive statement due to lack of data on sFLT-1 levels in study subjects.

The preeclamptic placenta are in hypoperfused and hypoxic state [23], mainly due to deficient spiral artery invasion by the trophoblasts. Resulting in failure of blood vessels to convert from high pressure low capacitance to low pressure high capacitance vessels, thus putting placenta at increased risk for development of acute atherosis and hypoxia with possible culmination in infarction and necrosis [24] and finally leading to abnormal placentation and cytokine release in the maternal circulation which damage the endothelium [25, 26]. VEGF is one of the cytokines which is released in maternal circulation during pregnancy and the increased concentration of VEGF is exaggerated by placental hypoxia resulting from vasospasm, hypertension, and increased vascular permeability.

In our study, we also found that serum total protein and albumin levels were significantly low in preeclamptic patients as compared to the control (*p* < 0.05). Preeclampsia is associated with increased capillary permeability resulting in endothelial damage which is also responsible for the observed proteinuria and low level of the serum total protein and albumin [27].

Increase urinary protein excretion observed during normal pregnancy is due to combination of increased glomerular filtration rate and increased permeability of the glomerular basement membrane [28].

We also found the elevated level of serum AST and creatinine (*p* < 0.05) in preeclampsia compared to control group. The elevated level of serum AST in preeclampsia is due to arteriolar spasm, involving the myocardium, liver, kidney, and brain and resulting tissue hypoxia.

Also, serum uric acid is significantly elevated in PIH subjects as compared to normal pregnant females (*p* < 0.05). As uric acid is a potent inhibitor of endothelial function, it induces the systemic and glomerular hypertension and passes freely into the fetal circulation [29].

## 5. Conclusion

So we conclude that serum levels of VEGF are increased in PIH subjects compared to normal pregnant female. While the association is clearly established by our study, lack of data on sFLT-1 levels restricts us to conclude the causal relationship. Thus, prospective studies with parallel sFLT-1 estimation are needed to establish causal relationship. Also this may be a possible junction which may pose a platform for planning clinical trials of various anti VEGF drugs in PIH.

## Figures and Tables

**Table 1:** Biochemical variables of PIH, normal pregnant, and control subjects.

Characteristics	PIH (*n* = 35) (Mean ± SD)	Normal pregnant (*n* = 35) (Mean ± S.D.)	Control (*n* = 20) (Mean ± SD)	*p* value
Age (Yrs)	24.37 ± 3.43	23.37 ± 2.83	24.25 ± 3.39	0.38
Rbs (mg/dL)	76.46 ± 10.8	76.71 ± 9.75	86.25 ± 14.5^*∗*#^	**0.01**
Urea (mg/dL)	10.74 ± 4.17	9.2 ± 3.34^*∗*^	17.35 ± 5.28^*∗*#^	**<0.0001**
Creatinine (mg/dL)	0.691 ± 0.131	0.617 ± 0.101^*∗*^	0.81 ± 0.2^*∗*#^	**0.0005**
Total bilirubin (mg/dL)	0.514 ± 0.718	0.451 ± 0.365	0.445 ± 0.167^*∗*^	0.16
Direct bilirubin (mg/dL)	0.168 ± 0.446	0.137 ± 9.727	0.145 ± 5.10^*∗*^	0.07
AST (IU/L)	32.23 ± 25.4	22 ± 7.69^*∗*^	17.65 ± 6.78^*∗*#^	**0.0032**
ALT (IU/L)	19.11 ± 11.9	12.17 ± 3.38^*∗*^	15.25 ± 4^#^	**0.0007**
Total protein (g/dL)	6.374 ± 0.5	6.349 ± 0.453	7.035 ± 0.677^*∗*#^	**0.0004**
Albumin (g/dL)	3.289 ± 0.306	3.346 ± 0.167	4.115 ± 0.344^*∗*#^	**<0.0001**
Calcium (mg/dL)	8.703 ± 0.863	8.67 ± 0.396	9.32 ± 0.795^*∗*#^	**0.001**
Uric acid (mg/dL)	4.166 ± 1.18	3.53 ± 1.04^*∗*^	4.02 ± 1.27	0.16
Sodium (mmol/L)	135.6 ± 3.38	135.7 ± 2.77	137.5 ± 1.64^*∗*#^	**0.02**
Potassium (mmol/L)	3.602 ± 0.375	3.597 ± 0.33	3.8 ± 0.249^#^	0.14

^*∗*^ *p* < 0.05 versus PIH group.

^#^ *p* < 0.05 versus normal pregnant group.

**Table 2:** Levels of serum VEGF in PIH, normal pregnant, and control subjects.

Characteristics	PIH (*n* = 35) Median (min–max)	Normal pregnant (*n* = 35) Median (min–max)	Control (*n* = 20) Median (min–max)	*p* value
Serum VEGF (pg/mL)	109.19 (3.38–619)	20.82 (1.77–619)^*∗*^	4.92 (1.13–13.07)^*∗*#^	<0.0001

^*∗*^ *p* < 0.05 versus PIH group.

^#^ *p* < 0.05 versus normal pregnant group.
